# Human Adipose-Derived Mesenchymal Stem Cells-Incorporated Silk Fibroin as a Potential Bio-Scaffold in Guiding Bone Regeneration

**DOI:** 10.3390/polym12040853

**Published:** 2020-04-07

**Authors:** Dewi Sartika, Chih-Hsin Wang, Ding-Han Wang, Juin-Hong Cherng, Shu-Jen Chang, Gang-Yi Fan, Yi-Wen Wang, Chian-Her Lee, Po-Da Hong, Chih-Chien Wang

**Affiliations:** 1 Laboratory of Adult Stem Cell and Tissue Regeneration, National Defense Medical Center, Taipei 114, Taiwan; dd.sartika@gmail.com (D.S.); belle661011@gmail.com (S.-J.C.); u9310318@gmail.com (G.-Y.F.); 2 Department of Plastic and Reconstructive Surgery, Tri-Service General Hospital, National Defense Medical Center, Taipei 114, Taiwan; super-derrick@yahoo.com.tw; 3 Department of Dentistry, School of Dentistry, National Yang-Ming University, Taipei 112, Taiwan; nn2399906@ym.edu.tw; 4 Department and Graduate Institute of Biology and Anatomy, National Defense Medical Center, Taipei 114, Taiwan; i72bbb@gmail.com (J.-H.C.); christmas1035@hotmail.com (Y.-W.W.); 5 Department of Gerontological Health Care, National Taipei University of Nursing and Health Sciences, Taipei 112, Taiwan; 6 Division of Rheumatology/Immunology/Allergy, Department of Internal Medicine, Tri- Service General Hospital, National Defense Medical Center, Taipei 114, Taiwan; 7 Division of Urology, Department of Surgery, Tri-Service General Hospital, National Defense Medical Center, Taipei 114, Taiwan; 8 Department of Orthopedics, Taipei Medical University Hospital, Department of Orthopedics, School of Medicine, College of Medicine, Taipei Medical University, Taipei 110, Taiwan; chianherlee@yahoo.com.tw; 9 Department of Materials Sciences and Engineering, National Taiwan University of Science and Technology, Taipei 106, Taiwan; poda@mail.ntust.edu.tw; 10 Department of Orthopedic Surgery, Tri-Service General Hospital, National Defense Medical Center, Taipei 114, Taiwan

**Keywords:** silk fibroin, scaffold, adipose stem cells, bone regeneration, calvarial defects, bone tissue engineering

## Abstract

Recently, stem cell-based bone tissue engineering (BTE) has been recognized as a preferable and clinically significant strategy for bone repair. In this study, a pure 3D silk fibroin (SF) scaffold was fabricated as a BTE material using a lyophilization method. We aimed to investigate the efficacy of the SF scaffold with and without seeded human adipose-derived mesenchymal stem cells (hASCs) in facilitating bone regeneration. The effectiveness of the SF-hASCs scaffold was evaluated based on physical characterization, biocompatibility, osteogenic differentiation in vitro, and bone regeneration in critical rat calvarial defects in vivo. The SF scaffold demonstrated superior biocompatibility and significantly promoted osteogenic differentiation of hASCs in vitro. At six and twelve weeks postimplantation, micro-CT showed no statistical difference in new bone formation amongst all groups. However, histological staining results revealed that the SF-hASCs scaffold exhibited a better bone extracellular matrix deposition in the defect regions compared to other groups. Immunohistochemical staining confirmed this result; expression of osteoblast-related genes (*BMP-2*, *COL1a1*, and *OCN*) with the SF-hASCs scaffold treatment was remarkably positive, indicating their ability to achieve effective bone remodeling. Thus, these findings demonstrate that SF can serve as a potential carrier for stem cells, to be used as an osteoconductive bioscaffold for BTE applications.

## 1. Introduction

Bone defects, caused by diseases or traumas, are still clinically challenging. With advancing age, they can lead to a life-long disability in many patients [1]. Successful treatment of bone defects depends on the interaction between the injured area and the implant such that any missing piece of bone is replaced with a proper functional substitute. Despite the advantages of bone repair techniques including autologous bone grafting, xenografts, rigid fixation, decortication, and microvascular free tissue transfer, problems such as immune rejection, failure, limited availability of materials, and significant morbidity are observed [2,3,4]. Bone tissue engineering (BTE) offers potential strategies for bone defect therapies and remains to gain clinical significance annually [5]. In BTE, the current trends focus on the development of degradable integrated 3D-scaffolds that mimic the properties of the natural bone extracellular matrix (ECM) and support the formation of new tissue/bone [6,7]. Hence, the strategies of functional temporary matrices including scaffolds, cells, growth factors, and their interrelation in the microenvironment are crucial and major concerns for the reconstruction of the defective bone.

Biopolymers containing natural ECM components (e.g., collagen, gelatin, silk, chitosan, alginate, and some polynucleotides) are widely known as potential scaffolds in BTE. ECM-based scaffolds have been suggested to closely resemble the original tissues and possess remarkable osteoinductive characteristics [8,9]. Silk fibroin (SF) produced by silkworm *B. mori* is a natural polymer composed of the amino acids glycine, alanine, serine, valine, and tyrosine; it represents a highly attractive scaffolding material suitable for tissue regeneration due to its biocompatibility, biodegradability, easy processability, and mechanical and thermal properties [10,11]. Moreover, SF is also less immunogenic and inflammatory compared to other reported biomaterials [12,13], and is widely used in BTE applications, as it possesses abundant β-sheet crystalline regions that act as nucleating sites to promote mineralization and hydroxyapatite deposition in native bone tissues [14]. In nature, crystal nucleation and growth are regulated by carboxylate-rich polypeptides, which are present within a macromolecular matrix with organized β-sheet domains. The β-sheet peptides comprise a hydrophobic tail linked to a hydrophilic amino acid sequence [15]. Thus, charged amino acids are considered as effective sequences to provide nucleation sites, where calcium ions are gathered and then attract phosphate ions through electrostatic interaction, leading to biological mineralization [16,17].

The stimulation of natural behavior by way of osteogenesis, osteoinduction, and osteoconduction, as well as vasculogenesis and angiogenesis is key to bone healing. In these associated mechanisms, the presence of osteoblastic cells is vital as they are involved in generating the bone surface and participate in the production of ECM proteins that are important for phosphate metabolism and mineralization [18]. Stem cells are the primary source of cells that can be harvested and induced to differentiate into osteoblasts, either in vitro or in vivo. The best clinical option of stem cell-based BTE is the use of mesenchymal stem cells (MSCs) as they have minimal ethical concerns and a risk of teratoma formation [19,20]. Human adipose-derived mesenchymal stem cells (hASCs) appear to offer the same differentiation potential as MSCs; further, they are abundant in the body and are easy to isolate. Previous studies have demonstrated the osteogenic differentiation potential of hASCs when cultured in various scaffolds from inorganic and organic sources, showing that this combination successfully improved the osteogenic performance, accelerated the vascularization of the implanted area, promoted matrix mineralization, and improved bone formation [21,22,23,24]. Since hASCs are multipotent, available in large quantities per individual, and transplantable, they can be an ideal cell source for bone regeneration treatment.

A better understanding of the physiology, osteogenic potential, and differentiation mechanisms of hASCs-combined scaffolds has allowed the continuous development of innovative strategies in designing bone tissue engineering products. In the present study, we aimed to fabricate a pure 3D SF scaffold using a simple lyophilization method based on temperature modulation. The novelty of scaffold fabrication lies in the use of a pure SF solution by avoiding harsh crosslinking agents or any additional materials in order to entirely expose the SF properties. To our knowledge, the most currently used methods of SF scaffold preparation are combined with other organic or inorganic materials. Further, we seeded hASCs on the SF scaffold and investigated the effectiveness of the proposed combination for treating critical bone defects and examining its underlying mechanism in guiding bone regeneration.

## 2. Materials and Methods 

### 2.1. Preparation of Silk Fibroin (SF) Scaffold

#### 2.1.1. SF Solution Preparation

**Figure 1:**
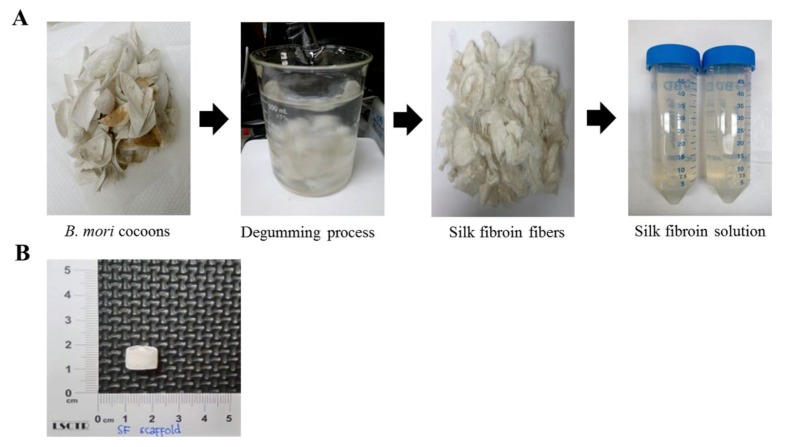
Preparation of silk fibroin (SF) scaffold. (**A**) Preparation of SF solution; (**B**) macroscopic appearance of the SF scaffold fabricated by a lyophilization method.

Silk cocoons of *Bombyx mori* were purchased from the Danee Company, New Taipei City, Taiwan. Briefly, the cocoons were cut into pieces and the sericin coating was removed by degumming with a 0.02 M Na_2_CO_3_ solution at 90–100 °C for 30 min. Then, the silk fibers were washed with distilled water and dried overnight at 37 °C. After that, the degummed fibers were dissolved in a 9.3 M LiBr solution (20% *w/v* solution) at 60 °C for 4 h, and dialyzed against distilled water using a dialysis tube (Cellu·Sep®, molecular weight cut-off: 3500), followed by centrifugation at 10,000× *g* for 20 min. The final concentration of the SF solution was about 6 wt %, determined by weighing the remaining solid after drying at 60 °C. The SF solution was stored at 4 °C until use (Figure 1A).

#### 2.1.2. SF Scaffold Fabrication

The pure 3D SF scaffold was fabricated by a lyophilization method based on temperature modulation (Figure 1B). The SF solution (1 mL) was first loaded into a vial and gradually cooled for overnight freezing at a rate of 2 °C min^−1^ until the samples reached −20 °C. Then, the frozen SF solution was transferred into a freeze-drying chamber (FD24-4S; Kingming, Taipei, Taiwan) at −20 °C under a 300 mbar vacuum for 48 h, and was gradually ramped to 25 °C. Further, the SF scaffold was treated with a 75% ethanol solution for 30 min, and the lid was then removed to allow solvent evaporation.

### 2.2. Physical Characteristics

#### 2.2.1. Fourier Transform Infra-Red (FTIR) Spectroscopy

The structure of the SF scaffold was analyzed by FTIR spectroscopy on a Nicolet 8700 Research spectrometer (Thermo Scientific, Waltham, MA, USA) equipped with a MIRacle™ attenuated total reflection (ATR) Ge crystal cell in the reflection mode and mercury-cadmium-telluride (MCT) as the infrared detector. For each measurement, 32 scans were coded with a resolution of 4 cm^−1^, and wave number ranging from 400–4000 cm^−1^.

#### 2.2.2. Scanning Electron Microscopy (SEM)

The surface morphology of the SF scaffold was examined using a Hitachi S–3000N SEM (Hitachi High Technologies, Krefeld, Germany). The samples were attached to carbon stubs and mounted on aluminum stubs. SEM images were acquired at an accelerating voltage of 1.5 kV, a working distance of ~15.0 mm, and 100×–500× magnification. The diameter and porosity of SF scaffolds were measured based on SEM micrographs using the ImageJ software (National Institutes of Health, Bethesda, MD, USA; n = 5).

### 2.3. Cell Viability

The SF scaffold was placed in direct contact with a cell culture to assess its biocompatibility. Human adipose-derived mesenchymal stem cells (hASCs) provided by Dr. Cherng were cultured in a keratinocyte serum-free medium (KSFM; Life Technologies Ltd., Paisley, Scotland, UK) supplemented with a 10% fetal bovine serum (FBS; Hyclone, Logan, UT, USA) at 37 °C in humidified air containing 5% CO_2_. The scaffolds were sterilized overnight by UV irradiation, followed by hASCs seeding onto the scaffolds at a density of $1\times 10^{6}$ cells cm^-2^ and incubating under 5% CO_2_ at 37 °C. The culture medium was replaced every two days during incubation. Cell viability was analyzed by the 3- (4, 5-dimethylthiazol-2-yl) -2, 5-diphenyltetrazolium bromide colorimetric (MTT) assay. Cells incubated without the scaffold served as the control. After two weeks of incubation, the medium from each group was removed and replaced with the MTT solution (5 mg mL^−l^) and incubated at 37 °C for 4 h. Then, the supernatant was carefully removed and the dimethyl sulfoxide solution was added to dissolve the crystals by gentle agitation for 10 min. The absorbance of each group at 570 nm was read on a microplate reader (Bio-Tek ELX-800; BioTek, Winooski, VT, USA). The tests were performed in triplicate.

### 2.4. In Vitro Osteogenic Differentiation

First, SF scaffolds were sterilized overnight by UV irradiation, followed by hASCs seeding onto the scaffolds at a density of $1\times 10^{6}$ cells cm^−2^ and incubating under 5% CO_2_ at 37 °C in KSFM supplemented with 10% FBS. The next day, the medium was replaced with an osteo-inductive medium consisting of Dulbecco’s modified Eagle’s medium, 10% FBS, 0.1 μM dexamethasone, 50 μM ascorbate-2-phosphate, 10 mM β-glycerophospate, and 1% penicillin/streptomycin (all from Sigma-Aldrich; Merck, Darmstadt, Germany). The culture medium was replaced every two days, for one and two weeks of incubation.

#### 2.4.1. Alizarin Red Staining

Alizarin red staining was performed to examine the presence of calcium deposits on the extracellular matrix indicated by the formation of red nodules. This formation of mineralized nodules marks the mature differentiation of osteoblasts and represents a morphological manifestation of osteoblast function. After one and two weeks of incubation, the SF-hASCs scaffolds were fixed with a 0.1% acetic acid solution for 30 min, washed twice with a phosphate-buffered saline, and kept overnight at 4 °C. Then, the SF-hASCs scaffolds were immersed in the post-fixed solution containing 4% para-formaldehyde-sucrose for 2–3 days. Further, the SF-hASCs scaffolds were cryosectioned at 50 μm. Then, the cryosectioned samples were fixed with 4% para-formaldehyde for 30 min and rinsed with distilled water before staining with the Alizarin red solution (Sigma-Aldrich; Merck, Darmstadt, Germany) for 5 min. The stained samples were observed under a light microscope and photographed using a SPOT-RT digital camera (Diagnostic Instruments, Detroit, MI, USA). The nodule area was measured based on staining images using the ImageJ software (n = 3).

#### 2.4.2. Von Kossa Staining

Von Kossa staining was performed to examine the presence of phosphate deposits on the extracellular matrix indicated by the formation of black nodules. This formation of mineralized nodules marks the mature differentiation of osteoblasts and represents a morphological manifestation of the osteoblast function. Cryosectioned SF-hASCs scaffolds were stained using a Von Kossa kit (Abcam, Cambridge, UK) according to the manufacturer’s instructions. Then, the stained samples were observed under a light microscope and photographed with a SPOT-RT digital camera (Diagnostic Instruments, Detroit, MI, USA). The nodule area was measured based on staining images using the ImageJ software (n = 3).

### 2.5. Rat Calvarial Defect Model

Thirty male Sprague-Dawley rats, aged eight weeks (250–300 g), were purchased from Bio-LASCO Co. Ltd. (Taipei, Taiwan). The experimental protocol was reviewed and approved by the Institutional Animal Care and Use Committee (IACUC-17-068) at the National Defense Medical Center (Taipei, Taiwan). Rats were randomly divided into three groups of treatment: Lesion control, SF scaffold, and hASCs-SF scaffold. Rats were anaesthetized with an intraperitoneal injection of xylazine (8 mg/kg) and ketamine (100 mg/kg), followed by a subcutaneous injection of enrofloxacin (0.05 mg/kg) as microbial prophylaxis. After being shaved and sterilized, the skin and underlying tissues including the temporal muscle were detached to expose the calvarial bone. Calvarial bone defects were generated with the aid of a dental bur and two defects of 5 mm in diameter were generated per cranium. The calvarial defects were either left empty or surgically filled by putting the tested materials inside the defect. All rats undergoing surgery were kept warm with an electric blanket during the operation and for 3 h afterwards. Rats were kept in individual cages under aseptic conditions and were sacrificed at six and twelve weeks after surgery. Then, bone specimens were harvested for further evaluation.

### 2.6. Micro-Computed Tomography (Micro-CT)

Calvarial bones were radiographically imaged using micro-CT (SkyScan 1172; Bruker Micro-CT, Kontich, Belgium) and were scanned at a 65 kV/385 mA source voltage/current, with a 1-mm aluminum filter. The pixel size (resolution), rotation step, and exposure time were setup at 35 μm, 0.6° over 360°, and 400 ms, respectively. The micro-CT analysis was performed with the CTAN software (SkyScan; Bruker Micro-CT). Appropriate magnification was used throughout the observation, and the micrograph results were compared among groups; 3D images of the defect area were constructed using CTVol Software Version 2.6, for imaging rendering and visualization.

### 2.7. Histological Analysis

Samples were fixed with a 4% paraformaldehyde solution for three days and were decalcified with a 10% ethylenediaminetetraacetic acid (EDTA; Shanghai Chemical Reagent Co., Shanghai, China) for four weeks on a shaking table at room temperature. Afterwards, the samples were dehydrated in a 0.33 M sucrose buffer at 4 °C. Frozen sections (5 μm) of decalcified specimens were serially cut, parallel and perpendicular to the long axis of the femur, using a freezing microtome (Leica, Wetzlar, Germany). All sections were preserved at −20 °C. Then, the sections were stained with Hematoxylin and Eosin (H&E) and Masson’s trichrome (Sigma-Aldrich, Laborchemikalien GmbH, Hanover, Germany) according to the manufacturer’s instructions. The stained sections were observed using an inverted microscope (BX53, Olympus, Tokyo, Japan).

### 2.8. Immunohistochemistry

The sections were treated with 0.2% Triton X-100 (Sigma-Aldrich, Laborchemikalien GmbH, Hanover, Germany) for 15 min, washed thrice with a phosphate-buffered saline (PBS), blocked with a 10% normal goat serum, and incubated with a primary antibody for 2 h at room temperature. The primary antibodies used were bone morphogenetic protein-2 (BMP-2), collagen type I alpha 1 (COL1a1), and osteocalcin (OCN) (1:1000 dilution; all from Santa Cruz Biotechnology, CA). After washing thrice with PBS, the sections were incubated for 1 h at 27 °C with a secondary antibody, biotinylated IgG-conjugated antirabbit, antimouse or antigoat, depending on the primary antibody (1:1000 dilution; Vector Laboratories, Burlingame, CA). For visualization of the nucleus, the sections were incubated for 15 min with a 1:5000 dilution of Hoechst 33342 in PBS (Invitrogen, Thermo Fisher Scientific, Waltham MA, USA). Then, the sections were observed using an inverted microscope (BX53, Olympus, Tokyo, Japan). The positive staining area was quantified.

### 2.9. Data Analysis

Data are presented as the means ± standard error of the mean. All quantitative data were statistically compared and analyzed by one-way ANOVA. The significance in differences was denoted as ‘***’ for *p* ≤ 0.001, ‘**’ for *p* ≤ 0.01, and ‘*’ for *p* ≤ 0.05.

## 3. Results and Discussion

### 3.1. Characterization of SF Scaffold

**Figure 2:**
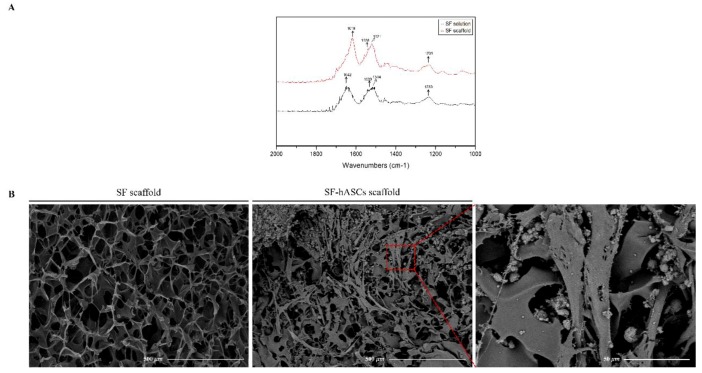
Characterization of silk fibroin (SF) scaffold. (**A**) FTIR spectra of SF; (**B**) morphology of SF scaffolds before and after seeding with human adipose-derived mesenchymal stem cells (hASCs).

FTIR was performed to examine the structural characteristics of the SF scaffold compared to the SF solution. As shown in Figure 2A, the SF solution displayed bands at 1642 cm^−1^ for amide I (C–O stretching), 1514 cm^−1^ with a shoulder at 1539 cm^−1^ for amide II (secondary N–H bending), and 1230 cm^−1^ for amide III (C–N and N–H functionalities) on the IR spectra, indicating a characteristic of SF with a primarily amorphous structure (random coil and/or α-helix conformations) [25,26]. After lyophilization, the SF scaffold displayed bands at 1619 cm^−1^ (amide I), 1521 cm^−1^ with a shoulder at 1538 cm^−1^ (amide II), and 1231 cm^−1^ (amide III). The band at 1642 cm^−1^ in the SF solution was remarkably observed to shift to 1619 cm^−1^ in the SF scaffold, representing the transition of random coil to β-sheet formation, as the lyophilization process and ethanol treatment increase the crystallinity and diminish the water solubility of the treated samples [27,28]. In addition to favorable mechanical properties, this transition makes SF more attractive as a biomaterial for BTE because the β-sheet structure can also act as a nucleating site to promote mineralization and cell adhesion capabilities [14,29].

A scaffold is a temporary 3D platform designed to mimic the ECM for guiding the healing process and promoting the regeneration of functional bone. Hence, the scaffold must be biocompatible and its microstructure should provide sufficient pore sizes and distribution with fine interconnectivity for nutrient and oxygen transport to cells, which leads to proper cell growth and attachment [30,31,32]. Additionally, the network structures of the pores also assist in guiding and promoting new tissue formation [33]. Thus, the morphology of the SF scaffold was investigated by SEM (Figure 2B). The surface of the SF scaffold displayed a uniformly porous and sponge-like structure. It consisted of pores ~170 μm in diameter and ~75% porosity with a high interconnection, which is considered suitable for cell growth and vascularization. Further, we incubated hASCs on the SF scaffold for seven days and used the SEM-captured image to depict the morphology of cells and cell adhesion to the scaffold. Our results showed that the cells adhered well on the SF scaffold, indicating that the SF scaffold is biocompatible and provides an excellent environment for cell attachment and function. 

### 3.2. In Vitro Biocompatibility and Osteogenic Differentiation

**Figure 3:**
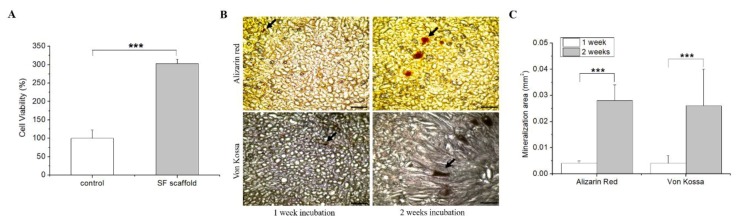
In vitro biocompatibility and osteogenic differentiation of hASCs seeded on the SF scaffold. (**A**) Cell viability analysis of SF scaffold; (**B**) Alizarin red and Von Kossa staining of the cryosection slices of SF-hASCs scaffolds (black arrow = mineral deposition; scale bar = 500 μm); (**C**) quantitative determination of mineralization area in the SF-hASCs scaffolds (***, *p* ≤ 0.001).

In clinical applications, cell viability is an important parameter for evaluating the scaffold capacity to generate successful cell-biomaterial constructs. Previous studies have demonstrated that cell viability and proliferation were promoted by SF [34,35]. In this study, the SF scaffold cytotoxicity was evaluated on cultured hASCs for two weeks. The result showed that the SF scaffold did not affect the viability of hASCs and significantly increased the proliferation of hASCs compared to the control (*p* < 0.001), indicating that the SF scaffold is nontoxic and does not negatively influence cell proliferation (Figure 3A).

ECM mineralization induced by cells seeded on a 3D scaffold is considered as the final product of an effective culture in BTE. Briefly, we seeded hASCs on the SF scaffold and subjected them to osteogenic induction and culture for one and two weeks. Alizarin red and Von Kossa staining were further performed to observe ECM mineralization through the deposition of calcium and phosphate. This formation of mineralized nodules marks the mature differentiation of osteoblasts and represents a morphological manifestation of the osteoblast function. The results showed that the mineral deposits, represented by red and black nodules, were significantly larger in the SF scaffold after two weeks of incubation (Figure 3B,C), indicating that the SF scaffold successfully facilitated the differentiation of hASCs into osteoblasts. Osteoblasts are specialized fibroblasts that secrete and mineralize the bone matrix [18]; therefore, their presence in the scaffold is essential for guiding bone regeneration.

### 3.3. Bone Regeneration in Rat Calvarial Defect Model

**Figure 4:**
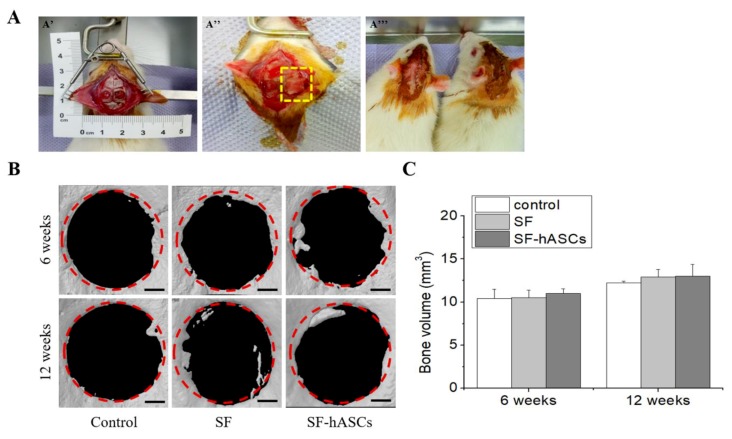
The rat calvarial defect model and micro-CT analysis at six and twelve weeks postimplantation. (**A**) Defects of 5 mm in diameter were created using a biopsy punch (A’); in one of these defects, the SF/SF-hASCs scaffold was inserted (yellow dash; A’’); postsurgery (A’’’); (**B**) micro-CT images of rat calvarial defects (scale bar = 1 mm); (**C**) bone volume results obtained from the quantitative analysis of micro-CT scans (n = 5).

Further, to assess the efficacy of the SF-hASCs scaffold for bone regeneration in vivo, we surgically created a critical calvarial bone defect in the rat model (5 mm in diameter) (Figure 4A). The defects were implanted with the SF-hASCs scaffold, SF scaffold only, or a blank that served as an untreated control. This implantation was well accepted by the local bone tissue without complications such as bleeding, infection, or other side effects in any of the cases, reflecting the outstanding biocompatibility of silk fibroin, which has long been used as an effective biomaterial. After six and twelve weeks of implantation, the micro-CT analysis was performed to examine the formation of a new mineralized bone in the total region of the initial defect. As observed, the newly formed bone is densely localized at the edges of the defect, particularly in the SF-hASCs group (Figure 4B). Typically, bone formation begins in the inner layer of the periosteum of the host bone near the injury site, gradually progressing towards the fracture gap [36,37]. Histomorphometric analysis results showed that the bone volume in the SF-hASCs scaffold treatment was the highest amongst all groups, attributed to the osteogenic differentiation of embedded hASCs in the SF scaffold for new bone formation (Figure 4C). However, no significant difference was observed. This can occur because the time point of less than twelve weeks is not sufficient for complete healing of the extreme calvarial defects [38,39] and the number of animals in each group was small. Thus, further studies with larger sample sizes and more time points are needed to clearly determine the differences between such groups.

### 3.4. Histological and Immunohistochemistry Analysis

As demonstrated by the micro-CT analysis, formation of mineralized bones is not significantly different amongst all groups; a qualitative assessment was further carried out to determine whether there were observable tissue, cellular, or molecular differences during the bone regeneration process. In pathological fractures or critical bone defects, bone regeneration is a complex trait; therefore, it is necessary to evaluate bone healing in the context of its quality and to determine how this treatment influences the microenvironmental activities during repair.

**Figure 5:**
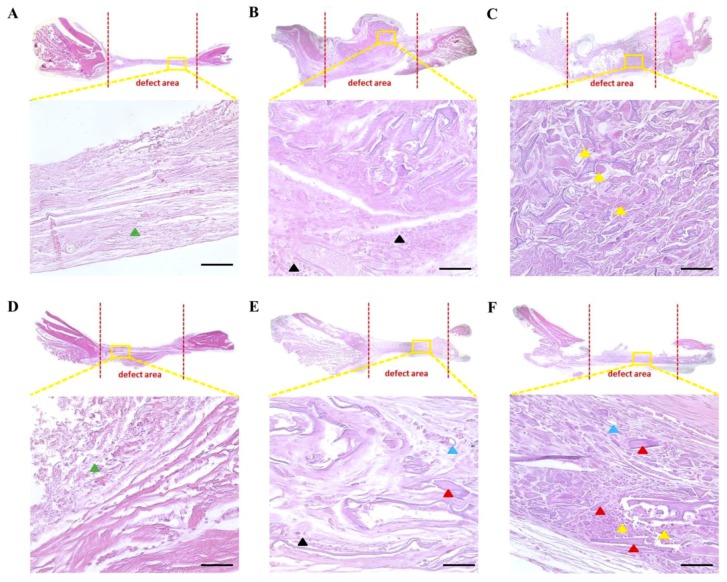
Hematoxylin and Eosin (H&E) staining of rat calvarial defects in the control, implanted SF scaffold, and implanted SF-hASCs scaffold groups. (**A**,**D**) Control group at six and twelve weeks postimplantation, respectively; (**B,E**) SF group at six and twelve weeks postimplantation, respectively; (**C,F**) SF-hASCs group at six and twelve weeks postimplantation, respectively (green triangle = fibrous connective tissue; black triangle = osteoblast; yellow triangle = osteocyte; red triangle = new bone; blue triangle = blood vessel; scale bar = 50 μm).

H&E staining revealed that the defect regions of the control group were covered with a fibrous connective tissue (green triangle) at six and twelve weeks postimplantation (Figure 5A,D). This condition is a consequence of the critical size defect, meaning that there is no spontaneous tissue regeneration [40]. A great number of osteoblasts (black triangle) within the defect region were observed in the SF group at six weeks postimplantation (Figure 5B), whereas, the SF-hASCs group displayed more osteocytes (yellow triangle) deposited at the same time (Figure 5C). Osteocytes are mature osteoblasts that co-concentrate for the new bone synthesis and have long been considered as quiescent bystander cells compared to osteoblasts and osteoclasts whose activities result in bone formation and loss [41]. However, osteocytes play an active role in blood calcium and phosphate ion homeostasis. A functional system of bone cells that serves in the differentiation from osteoblasts to osteocytes is important for maintaining the integrity and structure of the bone as a tissue [42,43]. This result indicated that the presence of hASCs in the SF scaffold boosted the transformation of osteoblasts into osteocytes, suggesting an acceleration of the bone remodeling process [44]. At twelve weeks postimplantation, new bone (red triangle) and blood vessels (blue triangle) were observed in the defect region of the SF group (Figure 5E); however, their presence was greater in the SF-hASCs group (Figure 5F). Moreover, more osteocytes were observed in the SF-hASCs group. 

**Figure 6:**
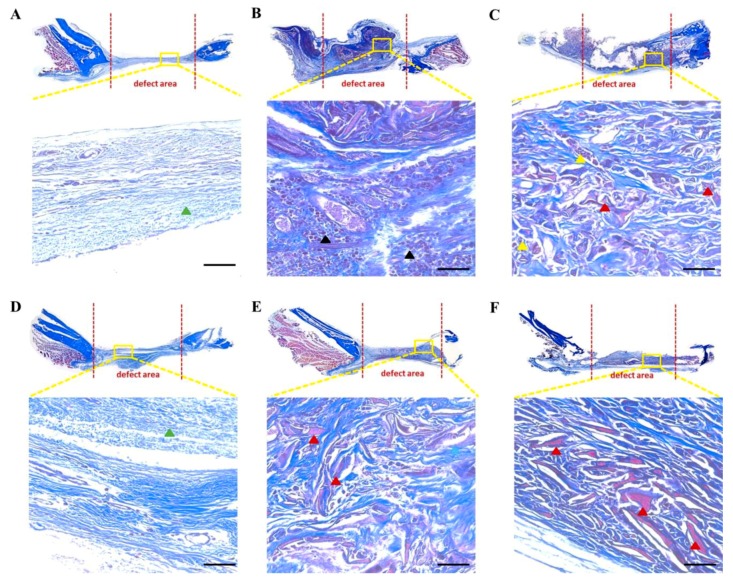
Masson’s trichrome staining of rat calvarial defects in the control, implanted SF scaffold, and implanted SF-hASCs scaffold groups. (**A,D**) Control group at six and twelve weeks postimplantation, respectively; (**B,E**) SF group at six and twelve weeks postimplantation, respectively; (**C,F**) SF-hASCs group at six and twelve weeks postimplantation, respectively (green triangle = fibrous connective tissue; black triangle = osteoblast; yellow triangle = osteocyte; red triangle = new bone; scale bar = 50 μm).

Masson’s trichrome staining further demonstrated that the defect regions of the control group were connected by a pale blue thin layer of fibrous connective tissues (Figure 6A,D). In contrast, the defect regions in the other two groups were connected by a densely red- and blue-stained new bone and some connective tissue, respectively, notably shown by the SF-hASCs group (Figure 6B,C,E,F). The dense blue color has a high specificity with collagen marking; thus, the results show that the SF and SF-hASCs groups have a higher amount of collagen inside the defect region compared to that in the control group. Since collagen deposition is one of the indicators of the onset of bone healing [45], the great amount of collagen in the SF and SF-hASCs groups indicates an immature extracellular matrix that will later be mineralized. On the contrary, the collagen observed in the control group is only a result of the formation of fibrous tissue caused by the critical size defect [40,45]. In addition, with time, a compact and more orderly new tissue was observed to form in the defect region of the SF-hASCs group compared to the SF group.

**Figure 7:**
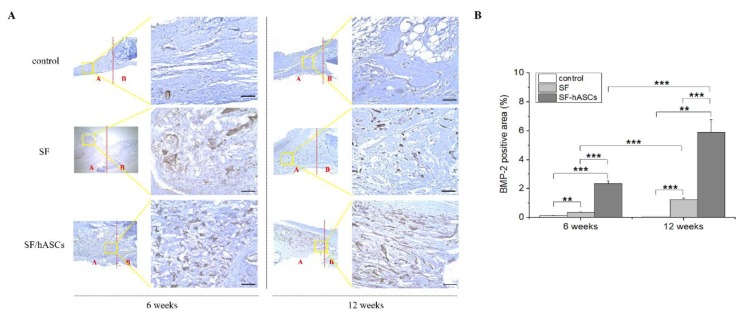
Bone morphogenetic protein-2 (BMP-2) immunohistochemical (IHC) staining of rat calvarial defects in the control, implanted SF scaffold, and implanted SF-hASCs scaffold groups. (**A**) IHC staining of BMP-2 positive cells, at six and twelve weeks postimplantation in three different groups. (**B**) Semi-quantitative analysis of the relative amounts of BMP-2-positive cells in three different groups (**, *p* ≤ 0.01; ***, *p* ≤ 0.001; scale bar = 50 μm; red A and B = defect area and host bone area, respectively).

**Figure 8:**
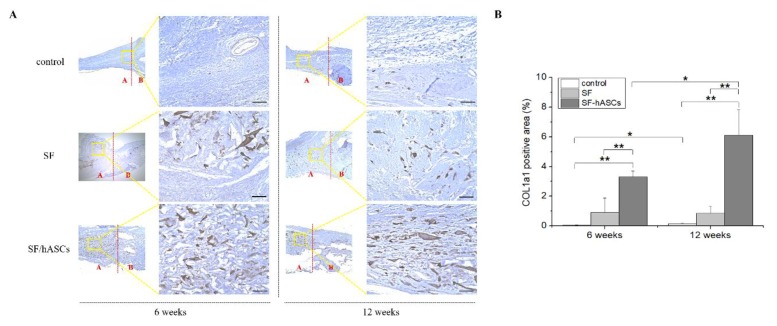
Collagen type I alpha 1 (COL1a1) immunohistochemical (IHC) staining of rat calvarial defects in the control, implanted SF scaffold, and implanted SF-hASCs scaffold groups. (**A**) IHC staining of COL1a1 positive cells at six and twelve weeks postimplantation in the three different groups. (**B**) Semi-quantitative analysis of the relative amounts of COL1a1-positive cells in the three different groups (*, *p* ≤ 0.05; **, *p* ≤ 0.01; scale bar = 50 μm; red A and B = defect area and host bone area, respectively).

**Figure 9:**
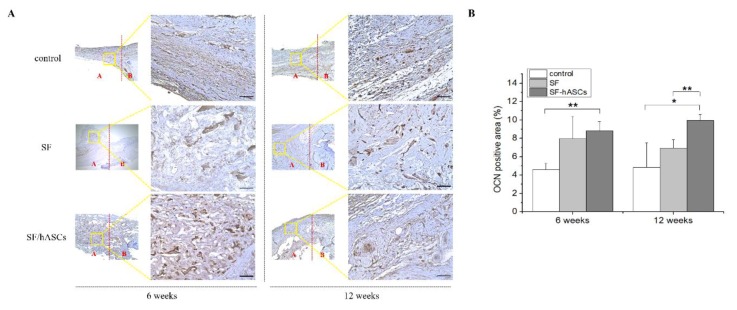
Osteocalcin (OCN) immunohistochemical (IHC) staining of rat calvarial defects in the control, implanted SF scaffold, and implanted SF-hASCs scaffold groups. (**A**) IHC staining of OCN positive cells at six and twelve weeks postimplantation in the three different groups. (**B**) Semi-quantitative analysis of the relative amounts of OCN-positive cells in the three different groups (*, *p* ≤ 0.05; **, *p* ≤ 0.01; scale bar = 50 μm; red A and B = defect area and host bone area, respectively).

Subsequently, immunohistochemical (IHC) analysis was performed to explore the quality of newly formed tissues in the area of the defect. In addition, three samples of each IHC staining for each group were selected for the semi-quantitative analysis. The gene expression of three different osteoblast markers was compared: BMP-2, COL1a1, and OCN. The IHC results demonstrated a remarkably higher positive expression of all genes in the defect regions with the SF-hASCs group treatment compared to the SF and control groups (Figure 7A, Figure 8A and Figure 9A), which appeared to be significantly increased in the SF-hASCs group as time progressed (Figure 7B, Figure 8B and Figure 9B). These genes are considered the master genes required for osteoblast differentiation and bone formation. BMP-2 is well known as a growth factor in bone regeneration due to its particularly strong osteoinductive function, and is involved in bone formation, bone remodeling, bone development, and osteoblast differentiation [46,47]. Type I collagen is mainly expressed by osteoprogenitors that undergo a proliferative stage as they initiate production and maturation of osteogenic ECM at the outset of bone repair [48]. Finally, they further expressed OCN during the mineralization of bone ECM. These findings are consistent with the histological data and indicate that the SF-hASCs scaffold can enhance the osteogenesis process during bone regeneration.

Previous studies have reported the osteogenic abilities of stem cells-incorporated SF to stimulate new bone formation using different scaffold designs and animal models [35,49,50]. Yan et al. demonstrated that, besides mediating cell adhesion, the RGD ligands incorporated within their SF-RGD gel promoted the osteogenic differentiation of bone marrow-derived mesenchymal stem cells encapsulated within the gel matrix, leading to bone regeneration in a mouse calvarial defect model, compared with a blank SF gel [35]. Further, the functionalized SF-GO-BMP-2 composite scaffold displayed good biocompatibility and excellent proliferation effects and biological activity, thus improving bone regeneration in critical-sized bone defects and enhancing the osteogenic differentiation of BMSCs [49]. Ye et al. revealed a high biocompatibility and osteoinduction of the SF/CS/nHA composite scaffold in the repair of a defect in the rabbit radial bone [50]. Further, the degradation rate of the scaffolds was compatible with the regeneration of bone tissues. All these data, along with our findings, suggest that the combination of stem cells and SF may indeed be an excellent functional bioscaffold for guiding bone formation.

## 4. Conclusions

In summary, this study has shown that the hASCs-incorporated SF scaffold had superior biocompatibility and osteogenic abilities to promote bone regeneration in critical rat calvarial defects. The presence of hASCs in the SF scaffold demonstrated an effective bone remodeling process as they boosted the transformation of osteoblasts into osteocytes and exhibited an improved bone extracellular matrix in the area of the defect. Thus, SF could serve as a potential carrier for stem cells to be used as an osteoconductive bioscaffold for BTE applications.
